# Ecosystem carbon storage in forest fragments of differing patch size

**DOI:** 10.1038/s41598-017-13598-4

**Published:** 2017-10-13

**Authors:** Lei Ma, Chunyu Shen, Duo Lou, Shenglei Fu, Dongsheng Guan

**Affiliations:** 1 Laboratory of Geospatial Technology for the Middle and Lower Yellow River Regions, College of Environment and Planning, Henan University, Kaifeng, 475004 China; 2 School of Environmental Science and Engineering of Sun Yat-sen University, No. 135, Xingang Xi Road, Guangzhou, 510275 P. R. China

## Abstract

Forest fragmentation threatens the ecosystem carbon (C) storage. The distribution patterns of ecosystem C density are poorly documented for fragmented forests of differing patch size. The objectives of this study were to examine C density in these forest ecosystems and the influence of edge effects on C density. Allometric equations were used to quantify aboveground biomass. Carbon density was estimated by analyzing the C concentration of each component. We found that ecosystem carbon density ranged from 173.9 Mg ha^−1^ in the small sized forest fragments, to 341.1 Mg ha^−1^ in the contiguous evergreen sub-tropical forest. Trees (46.5%) and mineral soil (50.2%) were the two largest contributors to the total ecosystem C pool in all fragments. Both C and nitrogen (N) in soil and fine roots were highly heterogeneous among the different fragment sizes and soil depths. We concluded that ecosystem C density of forest fragments were significantly influenced by patch size and edge effects. The fragmented forests in southern China play an important role in the C budget, and need urgent conservation. These results are likely to be further integrated into forest management plans and generalized into other contexts, to evaluate C stocks at the landscape scale.

## Introduction

Globally, forests and their associated soils store an estimated 45% of all available carbon (C) and contribute approximately half of the total terrestrial net ecosystem production^1^. Forests contain about 90% of the C stored in terrestrial vegetation and account for about 40% of the carbon exchange between the atmosphere and earth^2^. Forest C stocks are dynamic, being influenced by changes inland use, harvesting, natural disturbances, climatic stressors, and vegetation growth. Forests play a key role in global biogeochemical cycles, in particular the C cycle. To quantify the size of the terrestrial C pool, the amount of, and changes to, total tree biomass needs to be measured. Forest dynamics, and the related C storage, play an important role atmospheric CO_2_ concentrations^3^. Slight changes in the forest C pool could have important impacts on the global C balance. Consequently, accurate information concerning the quantity of biomass and C storage in forest ecosystems is needed to improve our understanding of the global C cycle^4^.

During the past two decades, there have been many studies that have estimated regional forest C storage and national C budgets^5–7^, including in China^8,9^. However, few of the studies have examined how forest patch size influences C amounts^10^. Patch size is relevant because it has a significant effect on many of the variables affecting forest ecosystem C storage (e.g., community structure, species composition)^11,12^. To better understand forest restoration as an option for atmospheric CO_2_ fixation, and to assess the effect of patch size on forest C storage, C pools and their variability need to be measured.

In the absence of disturbance, intact tropical forests likely act as C sinks, whereas fragmented forests may be vulnerable to C losses and accelerated C cycling^13^. Using remote sensing, Pütz^10^ estimated long-term C loss due to fragmentation in Neotropical forests. Pütz concluded that tropical forest fragmentation increased C loss and should be accounted for when attempting to understand the role of vegetation in the global C budget. In central and south America, major biomass losses occur in the immediate aftermath of fragmentation, resulting from the death of large, old-growth trees, especially close to fragment edges that are exposed to wind and fire^14^. These losses may be brought about by changes in habitat structure and tree species composition^15^.

Habitat fragmentation increases the amount of a forest edge exposed to other land uses. As the original habitat is progressively reduced into smaller and smaller patches, the fragments become increasingly isolated and affected by edge effects^3^. Edges are one of the critical elements affecting community composition in fragmented forest landscapes. Edges offer habitat for new species, while at the same time slowly diminishing the abundance of non-edge species^16^. As community composition, structure, and ecosystem biomass within the remnant patches changes, small fragments differ markedly in composition compared with the original forest, and species richness following fragmentation declines over time. Aboveground biomass of fragments can decline even further, due to the proliferation of relatively softer-wooded, shorter-statured pioneer species^3,17^. Wind and other abiotic factors can also change tree allometry, with trees in fragments being shorter for a given basal diameter^18,19^. Through these multiple processes, some with immediate effects and others acting more slowly (e.g., over species turnover and forest succession timescales), the C stock and C sequestration potential of fragmented forests may diminish over time.

The objective of our study was to quantify the effect of patch size on C pools (aboveground and belowground) in fragmented and intact forests. We compared fragmented Fengshui forests in Guangzhou, Guangdong Province, in southern China, with mature forest ecosystems in the Dinghushan Nature Reserve, also in Guangdong Province. We hypothesized that the forests in the Dinghushan Nature Reserve store relatively larger densities of C both in the vegetation and the soil compared with other sub-tropical forests in southern China^20^. This study examines C density within the two forests and addresses the following questions: (1) What is the C density of these forests, and how is it allocated across C pools?; (2) Is the C density in the fragmented forests affected by patch size?; and (3) How does the edge effect influence ecosystem C density in the Fengshui forests? The results of this study will increase our understanding of Fengshui forest ecosystems and help achieve their sustainability. This study will also contribute to global research on C loss due to forest fragmentation.

## Methods and Materials

### Study site

This study was undertaken in Guangzhou, Guangdong Province, southern China. Guangzhou lies between 22°26′N and 23°56′N, and 112°57′E and 114°03′E. The region is influenced by a typical sub-tropical monsoon climate. The annual mean temperature is 21.8 °C, and the annual precipitation is 1690 mm, of which more than 75% falls during the rainy season (March until the end of September)^21^. Typhoons and thunderstorms occasionally damage trees and the mild climate permits continuous vegetation growth throughout the year^21^. The Dinghushan Nature Reserve is located in the western part of Guangdong Province. It covers 1155 ha of low mountains and hilly landscapes and is characterized by a south sub-tropical monsoon climate with mean annual temperature of 20.9 °C. It is covered by well-protected sub-tropical and tropical monsoon evergreen broadleaved forests. The reserve has similar rainfall and temperature regimes as the forest fragments in Guangzhou^21^.

In rural areas of southern China, sub-tropical forest fragments can be found near local villages. These remnants are called Fengshui forests by local residents. These Fengshui forests are distributed widely in southernChina^16^. Fengshui forests have been protected by generations of villagers, usually for religious reasons. As a result, these forests have retained features of the original vegetation. These forest remnants provide a basis for examining biomass and C storage and testing the various theories of fragmentation in sub-tropical forests. Although the Fengshui forests occur near local villages, human disturbance has had no significant effect on most of the community characteristics^16^.

### Sample plot selection

The study was carried out between March and November, 2015. Fifteen fragmented (Fengshui) forests were selected in Guangzhou. Fragmented forests were selected in three fragment sizes: large (LF, ~35 ha), medium (MF, ~15 ha) and small (SF, ~5 ha), each with five replicates. There were five 40 m × 40 m quadrats in the core area of every fragmented forest patch. In addition, 25 40 m × 40 m quadrats were established in the Dinghushan Nature Reserve (DHS). These quadrats were spatially independent^21^ and used as our control to assess the importance of forest fragments in ecosystem C density in sub-tropical forest ecosystems.

In every 40 m × 40 m quadrat, all stems ≥1 cm diameter at 1.3 m height from the ground (diameter at breast height: DBH) were identified, and DBH (using a measuring tape) was recorded. Species identification was carried out by an experienced field botanist from the South China Botanical Garden, Chinese Academy of Sciences. Species abundance (woody species) and richness (woody, understory, and herbaceous species) were calculated as the individuals per species and total number of species, respectively. Total basal area (sum of π × (DBH × 0.5)^2^ for all individuals, m^2^ ha^−1^) and tree density (number of individuals per ha) were determined for each quadrat.

Wood samples were collected from randomly chosen individuals of each species (5 replicates). An increment borer was used to extract a 1-cm diameter core at a height of ~1.5 m on the main stem for each individual. Wood sample volume was quantified using water displacement, and dry mass was determined after at least 96 hour at 60 °C. Wood density (g cm^−3^) was calculated as the ratio of dry mass to fresh volume. Understory biomass was determined using destructive sampling techniques (i.e., total harvesting) for the shrub (DBH < 1 cm) and herbaceous layer, in five 2 m × 2 m sub-quadrats and five 1 m × 1 m sub-quadrats, respectively. For this study, we defined forest floor biomass as woody debris and surface litter above the mineral soil layer. These components were collected in five randomly selected 2 m × 2 m sub-quadrats within each 40 m × 40 m quadrat.

Soil samples were obtained from randomly selected vertical profiles. Ten 1 m deep soil profiles were dug per 40 m × 40 m quadrat in the DHS and forest fragments. After removing surface litter, we collected soil samples from seven soil depth intervals: 0–10 cm, 10–20 cm, 20–30 cm, 30–40 cm, 40–50 cm, 50–70 cm, and 70–100 cm. We used a soil bulk sampler with a 100 cm^3^, stainless steel cutting ring to collect samples from the seven soil intervals. Soil and roots were separated during initial sampling and processed independently. Roots were immediately placed into sealed polyethylene bags on ice in a cooler and then transported to the laboratory and stored in a freezer at −40 °C to ensure minimal damage to the live tissue and change in ion concentrations. Prior to processing in the lab, the roots were thawed and cleaned. Fine root biomass was only calculated for the uppermost 4 depth ranges because almost all (98.6%) of the fine roots were distributed in the first 0–40 cm of soil^22^. Soil samples from same soil depth intervals were mixed, sealed in plastic bags, and transported to the laboratory.

### Estimation of aboveground C pools

The aboveground C pool was comprised of C from the tree, understory live biomass, and forest floor. We calculated the C storage (Mg C ha^−1^) of living trees based on separate genus. The above ground tree biomass (AGTB) of trees was estimated using the allometric equation^23^:

1 $${\rm{AGTB}}={\rm{WD}}\times \exp [-1.499+2.148{\rm{lnD}}+0.207{({\rm{lnD}})}^{2}-0.0281{({\rm{lnD}})}^{3}]$$

where, WD is the wood density for each genus in g cm^−3^ and D is the diameter at 1.3 m in cm.

The understory layer and forest floor samples collected were oven dried at 80 °C to a constant weight for dry biomass determination. The total aboveground C stock was calculated by assuming that C content is 47.4% of the total biomass^24^.

### Estimation of belowground C pools

For each soil sample, soil bulk density was calculated as the ratio of soil dry weight to the soil cutting ring’s volume (i.e., 100 cm^−3^). The soil samples were air dried and samples that were collected from within the same plot were mixed and sieved to 0.15 mm, after removing plant materials. Soil C and N (Nitrogen), and root C and N concentrations were analyzed through combustion (Elementar vario MAX, Gwemany). The total ecosystem C pool consisted of aboveground biomass C, fine root C, and soil C.

### Edge effects measurements

In order to detect edge effects in the forest ecosystem, four transects (~100 m long) were established from the forest center to the edge, in each fragment. Five 20 m × 20 m quadrats (regular spacing) were established along each transect. In each quadrat, all stems and soil samples were also measured. Five topsoil (0–20 cm) samples were collected in each 20 m × 20 m quadrat with a 100 cm^3^ stainless steel cutting ring. Soil and fine roots were separated.

### Statistical analyses

The data on plant biomass, soil C and N, and fine root C and N concentrations within different quadrats and forest fragments were analyzed by one-way analysis of variance (ANOVA). The significant difference among different quadrats and forest fragments were evaluated at the 95% confidence level using the Duncan method. Arithmetic means ± standard errors (SE) are presented throughout the paper. In addition, linear regression was used to estimate the correlation between patch size and ecosystem C storage (aboveground biomass C density, fine root C density, and soil C density) and community characteristics (abundance and richness). Significance levels were set at P < 0.05 for all analyses. All statistical analyses were carried out in R version 3.1.2^25^.

## Results

### Stand characteristics of fragmented forests

A total of 134 tree species that belonged to 83 genera and 52 families were recorded from the 100 quadrats (75 in the fragmented forest and 25 in the DHS). The tree species richness was highest in the DHS (71 species), followed by LF (53 species) and MF (36 species). Patch size had a significantly positive correlation with species richness (R^2^ = 0.65, *P* < 0.05). Patch size also had a positive impact on basal area, with larger forest fragments having a larger basal area per hectare (R^2^ = 0.63, *P* < 0.05). Tree density was significantly (*P* < 0.05) greater in DHS than in the LF, MF, and SF (Table 1). Younger trees (DBH < 10 cm) made up the greatest proportion of trees among all forests. The proportion of trees in a forest decreased with increasing tree diameter. Larger trees (DBH > 40 cm) in the SF accounted for a greater proportion of all trees than in the MF, LF and DHS (Table 2).

**Table 1:** Community characteristics of fragmented forests with different patch size.

	Size (ha)		Richness	Basal area (m^2^ ha^−1^)	Tree density (stems ha^−1^)
SF	5.1 ± 0.1	a	22 ± 2 a	17.5 ± 0.7 a	1494 ± 99 a
MF	16.2 ± 0.9	b	36 ± 5 b	22.7 ± 0.5 b	1814 ± 102 b
LF	34.3 ± 1.8	c	53 ± 5 c	30.4 ± 0.4 c	2645 ± 127 c
DHS	1155 ± 0	d	71 ± 6 d	32.2 ± 0.3 d	3500 ± 70 d

SF stands for smaller patch size forest, MF stands for medium patch size forest, LF stands for larger patch size forest, and DHS stands for forest in the Dinghushan Nature Reserve. Richness refers to woody, understory, and herbaceous species. Duncan method was used to test the difference. Different letters indicate significant differences at *P* < 0.05.

**Table 2:** Proportion (%) of total stems by size class for each forest category.

	0–10 cm		10–20 cm		20–30 cm		30–40 cm		40–50 cm		>50 cm
SF	58.7 ± 1.4 a	A	16.1 ± 0.9 a	B	14.8 ± 0.8 a	B	9.7 ± 0.5 a	C	0.3 ± 0.1 a	D	0.4 ± 0.1 a D
MF	62.9 ± 1.5 b	A	13.7 ± 1.3 b	B	13.9 ± 0.6 a	B	8.9 ± 0.7 a	C	0.3 ± 0.1 a	D	0.3 ± 0.1 a D
LF	68.3 ± 1.3 c	A	12.7 ± 1.1 bc	B	11.6 ± 0.7 b	B	7.1 ± 0.5 b	C	0.2 ± 0.1 a	D	0.1 ± 0.1 b D
DHS	75.4 ± 0.9 d	A	11.1 ± 1.2 c	B	10.9 ± 0.5 b	B	2.3 ± 0.2 b	C	0.2 ± 0.1 a	D	0.1 ± 0.1 b D

SF stands for smaller patch size forest, MF stands for medium patch size forest, LF stands for larger patch size forest, and DHS stands for forest in the Dinghushan Nature Reserve. Duncan method was used to test the difference. Different letters indicate significant differences at *P* < 0.05. The lowercase letters are for fragments and uppercase letters are for stem size.

### Ecosystem C pools in the forests

Ecosystem C storage significantly varied among fragments with different patch size. A consistent patch size effect was observed. Across all 15 fragments sampled and the DHS, patch size was significantly correlated with ecosystem C storage (R^2^ = 0.74, P < 0.05). The C density of the biomass, fine roots, and soil decreased in size, with amounts in the DHS > LF > MF > SF (Table 3). Aboveground biomass (46.5%) and soil (50.2%) were the two largest contributors to the total C pool. Ecosystem C storage in the DHS was significantly (*P* < 0.05) greater than that of the LF, MF, and SF, respectively (Table 3).

**Table 3:** Total ecosystem C density (Mg ha^−1^) for forests of differing patch size

	Aboveground biomass C		Fine root C		Soil C		Ecosystem C	
SF	76.2 ± 9.7	a B	2.5 ± 0.4	a A	95.2 ± 5.8	a C	173.9 ± 15.9	a D
MF	116.2 ± 5.3	b B	9.8 ± 0.4	b A	115.8 ± 8.1	b B	241.8 ± 13.8	b C
LF	140.9 ± 11.5	c B	11.7 ± 0.5	c A	136.3 ± 5.3	c B	288.9 ± 17.6	c C
DHS	158.2 ± 3.2	d B	13.5 ± 0.5	d A	177.4 ± 6.8	d C	349.1 ± 10.5	d D

SF stands for smaller patch size forest, MF stands for medium patch size forest, LF stands for larger patch size forest, and DHS stands for forest in the Dinghushan Nature Reserve. Duncan method was used to test the difference. Different letters indicate significant differences at *P* < 0.05. The lowercase letters are for forest category and the uppercase letters are for different C storage groups.

Total soil C concentrations in the forests with different patch sizes are shown in Table 4. Soil C density in the DHS was significantly (*P* < 0.05) larger than that in the LF, MF, and SF, respectively. Patch size was significantly correlated with soil C storage (R^2^ = 0.73, *P* < 0.05). Soil C carbon density was highest in the uppermost soil layers (0–20 cm). Soil C density decreased rapidly from top soil layers (0–20 cm) to the 100 cm soil layer in every forest (Table 4). Soil C:N ratio decreased with the same pattern as soil C, decreasing from top soil layer to the 100 cm soil layer (Table 5). Fine root C density decreased from the top soil layers to the 40 cm soil layer in every fragment (Table 6). However, the fine root C:N ratio increased from the top soil layers to the 40 cm soil layer in every forest (Table 7). In addition, soil C values were positively correlated with fine root C density. Soil C exhibited a strong spatial correlation with fine root C density in each of the four soil layers (*P* < 0.05).

**Table 4:** Soil C stock (Mg ha^−1^) shown by depth increment for forests of differing patch size.

	SF		MF		LF		DHS	
Total	95.2 ± 5.6	a A	115.8 ± 8.1	a B	136.3 ± 5.3	a C	177.4 ± 6.8	a D
0–10 cm	34.2 ± 1.3	b A	46.9 ± 1.9	b B	69.3 ± 1.3	b C	81.9 ± 1.2	b D
10–20 cm	18.1 ± 2.0	c A	23.5 ± 1.7	c B	21.9 ± 1.2	c C	35.9 ± 2.2	c D
20–30 cm	12.2 ± 0.7	d A	14.7 ± 1.3	d B	17.1 ± 0.8	d C	19.8 ± 0.8	d D
30–40 cm	9.7 ± 0.3	e A	12.5 ± 1.5	d B	11.9 ± 0.5	e B	15.6 ± 1.1	e C
40–50 cm	7.6 ± 0.2	f A	9.8 ± 0.9	e B	8.7 ± 0.6	f B	11.7 ± 0.6	f C
50–70 cm	8.7 ± 0.7	e A	5.1 ± 0.4	f C	8.5 ± 0.4	f A	7.2 ± 0.5	g B
70–100 cm	4.7 ± 0.4	g B	3.3 ± 0.4	g A	4.9 ± 0.5	g B	5.3 ± 0.4	h B

SF stands for smaller patch size forest, MF stands for medium patch size forest, LF stands for larger patch size forest, and DHS stands for forest in the Dinghushan Nature Reserve. Duncan method was used to test the difference. Different letters indicate significant differences at *P* < 0.05. The lowercase letters are for depth and the uppercase letters are for forest category.

**Table 5:** Soil C:N ratio at different depths for forests of differing patch size.

	SF		MF		LF		DHS	
0–10 cm	8.1 ± 0.3	a A	10.3 ± 0.3	a B	12.2 ± 0.4	a C	13.8 ± 0.5	a D
10–20 cm	7.3 ± 0.3	b A	9.2 ± 0.3	b B	10.1 ± 0.4	b C	12.3 ± 0.4	b D
20–30 cm	6.3 ± 0.3	c A	7.8 ± 0.3	c B	8.6 ± 0.4	c C	10.5 ± 0.4	c D
30–40 cm	5.7 ± 0.2	d A	6.4 ± 0.4	d B	7.3 ± 0.3	d B	8.4 ± 0.3	d C
40–50 cm	4.9 ± 0.3	e A	5.6 ± 0.3	e B	6.5 ± 0.2	e B	7.7 ± 0.3	e C
50–70 cm	3.4 ± 0.2	f A	4.5 ± 0.2	f B	5.7 ± 0.3	f C	6.3 ± 0.5	f C
70–100 cm	2.9 ± 0.1	g A	3.4 ± 0.2.	g B	4.2 ± 0.2	g C	5.1 ± 0.3	g D

SF stands for smaller patch size forest, MF stands for medium patch size forest, LF stands for larger patch size forest, and DHS stands for forest in the Dinghushan Nature Reserve. Duncan method was used to test the difference. Different letters indicate significant differences at *P* < 0.05. The lowercase letters are for depth and the uppercase letters are for forest category.

**Table 6:** Carbon density (Mg ha^−1^) in fine roots, shown by mineral soil depth increment, for forests of contrasting patch size.

	0–10 cm		10–20 cm		20–30 cm		30–40 cm	
SF	1.1 ± 0.1	a A	0.8 ± 0.1	a B	0.4 ± 0.1	a C	0.1 ± 0.1	a D
MF	4.9 ± 0.1	b A	3.2 ± 0.1	b B	1.4 ± 0.1	b C	0.3 ± 0.1	a D
LF	6.7 ± 0.1	c A	3.2 ± 0.1	b B	1.6 ± 0.1	b C	0.2 ± 0.2	a D
DHS	7.8 ± 0.1	d A	3.4 ± 0.1	b B	1.9 ± 0.1	c C	0.4 ± 0.2	a D

SF stands for smaller patch size forest, MF stands for medium patch size forest, LF stands for larger patch size forest, and DHS stands for forest in the Dinghushan Nature Reserve. Duncan method was used to test the difference. Different letters indicate significant differences at *P* < 0.05. The lowercase letters are for forest category and uppercase letters are for depth.

**Table 7:** Fine root C:N ratio at different depths for forests with different patch size.

	0–10 cm		10–20 cm		20–30 cm		30–40 cm	
SF	51.7 ± 0.5	a A	53.5 ± 0.4	a B	55.8 ± 0.5	a C	56.9 ± 0.5	a D
MF	50.2 ± 0.3	b A	52.1 ± 0.2	b B	53.7 ± 0.4	b C	55.4 ± 0.4	b D
LF	47.8 ± 0.2	c A	49.5 ± 0.3	c B	51.6 ± 0.4	c C	53.4 ± 0.2	c D
DHS	46.9 ± 0.3	d A	47.2 ± 0.3	d A	49.3 ± 0.2	d B	51.7 ± 0.3	d C

SF stands for smaller patch size forest, MF stands for medium patch size forest, LF stands for larger patch size forest, and DHS stands for forest in the Dinghushan Nature Reserve. Duncan method was used to test the difference. Different letters indicate significant differences at *P* < 0.05. The lowercase letters are for forest category and the uppercase letters are for depth.

### Edge effects on ecosystem C pool

**Figure 1:**
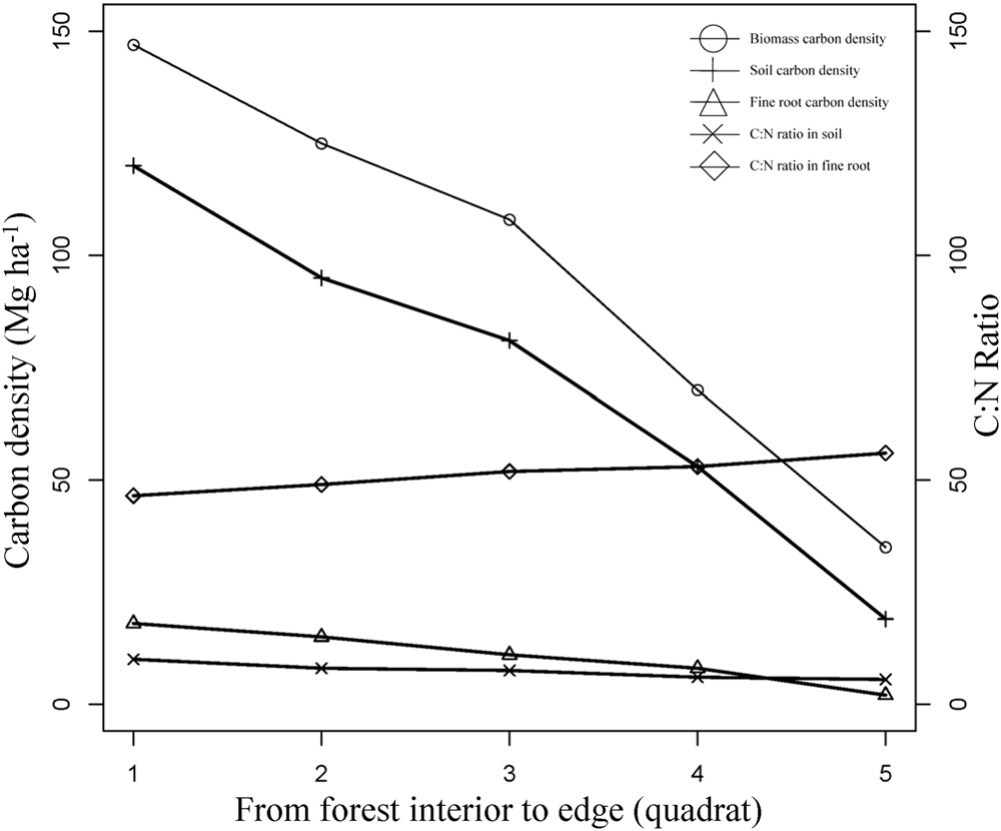
Edge effects on ecosystem (biomass, soil, and fine root) carbon density and C:N ratio in soil and fine root.

Aboveground biomass C, soil C, and fine root C density showed the same decreasing trend from the forest interior to the edge (Fig. 1). Ecosystem C density near the forest edge was very low compared with that in the core area of the forests. Aboveground biomass C had a positive relationship with soil C density. In addition, Soil C:N ratio decreased from the forest interior to the edge, while the fine root C:N ratio increased (Fig. 1).

## Discussion

This study is one of the first to empirically and explicitly test the relationship between patch size and C storage in woody ecosystems in a sub-tropical forest, using sites with similar plant community composition. It is also the first study to use rigorous field data to show the existence of edge effects on fine root C and N, in fragmented sub-tropical forests. Although our study did not involve manipulative experiments, and therefore causation cannot strictly be claimed, our results show clear trends that can be discussed in the light of current theories.

### Patch size effect

Patterns in forest biomass and soil C density are important quantitative aspects of ecosystems. The DHS had the greatest C density, followed by the LF. Our estimates of ecosystem C density of fragments demonstrated that ecosystem C density increased as the patch size of the stands increased. However, it is likely that patch size does not directly cause this result. Rather, patch size is related to variation in edge effect, which is further related to the characteristics of the community (i.e., species composition and community structure). Our results were consistent with those of other studies in sub-tropical forests, finding that fragment size has a marked influence on species composition and community structure^16^. Species composition and community characteristics were different among forest fragments of differing patch size. The strong effect of patch size on community structure, likely related to the abundance of pioneer species in small forest fragments, can lead to a rapid change over time in forest structure, species composition and ecological functionality^26–28^. Small sized fragments contain the smallest basal area recorded, a consequence of a simpler forest structure, characterized by a low abundance of trees. The changes in forest structure resulting from small-sized fragments have negatively impact on species that are dependent on specific characteristics in the forest structure^16^. Another study found that small forest fragments suffer substantial structural changes, as well as biomass and biodiversity loss in the long term^29^. Nascimento and Laurance^12^ analyzed the effects of fragmentation on community structure and found higher densities of pioneer species in fragments. Structural changes in the vegetation (e.g., higher density of trees with thinner trunks, larger proportion of pioneer species with lower wood density), decrease the C residence time in wood and accelerate decomposition, thereby shifting the C flow from necromass to the soil. The observation that Fengshui forest fragments had smaller ecosystem C density, indicates that the forests are influenced by patch size and have a greater potential to accumulate biomass.

Many studies have been conducted in fragmented forest ecosystems, either planted or natural, to test the determinants of ecosystem C storage^20,30^. However, there is no study, to our knowledge, that has quantified the effect of patch size on ecosystem C storage, especially soil and fine root C density, in a sub-tropical forest. We found that patch size was significantly correlated with soil C storage. All of the forest fragments analyzed in this study shared very similar climatic and soil conditions, leading to relatively small differences across these quadrats. Previous studies undertaken in the Fengshui forests revealed that humans had no significant effect on most of the community characteristics (e.g., abundance, richness, number of individuals within different DBH ranges)^16^.

### Edge effects on ecosystem C density

Our results showed that ecosystem C (i.e., aboveground biomass C, soil C, fine root C) density demonstrated edge effects. Forest fragments contain more edges exposed to other habitats than intact forests, and therefore could show more edges effects^31^. Carbon stocks are susceptible to edge effects, with reduced C density at edges compared to forest interiors, and with particularly strong impacts on the C value in smaller forest fragments. Due to temperature increases at the forest edge, more soil organic matter decomposes, leading to a reduction in soil C stock in these areas^29^. The differences observed in soil C storage between the core forest area and the fragment edges must be due to increases in the litter decomposition rate at the edges, as suggested by Nascimento and Laurance^12^.

Habitat fragmentation, and the resultant permanent forest edges, reduce forest capacity for C retention. This is because forest edges and fragments (i.e., edge-affected habitats) retain only half as much C as forest interior habitats^30–32^. These differences may be explained by changes in: species composition, the quality and quantity of litter, aboveground and belowground biomass, and the basal area of trees^33,34^. In addition, forests edges can be thought of as buffer zones over which environmental conditions progressively change with distance^11,26^, significantly impacting aboveground biomass C density. In this regard, our findings support the notion that the objectives of conserving biodiversity and mitigating climate change through C storage could be mutually achieved^35,36^.

### Soil C and N, fine root C and N

We measured the distribution of soil C and fine root C density across isolated forest fragments of differing patch size. Our results indicated that the distribution of soil and fine root C density were affected by patch size and showed edge effects. The reason for the observed differences in C density may be a function of the effect of patch size on aboveground biomass. Soil C and fine root C density are significantly affected by community characteristics, such as species diversity and community structure, which are influenced by patch size. Another study revealed that soil C density is spatially structured, partially due to the soil conditions that determine the decomposition rates of soil organic matter, but also due to the sink-source balance of the canopy structure and composition^34^.

Several studies have indicated that fine roots are the main contributors to soil C storage^22,37^. Our results showed that the distribution of soil C was closely associated with the distribution of fine roots, reinforcing their role as a important contributors to C stocks in mineral soils. There is general agreement that more than half of soil C is stored in the top 30 cm of soil^32,38^. At these shallow depths, fine root turnover contributes significantly to humus into the soil and is important for soil C accumulation^22^. In addition, N is also an important factor influencing soil C accumulation. Previous studies have shown that the rate of C accumulation is controlled by the rate of N accumulation^39^. Our results revealed that soil C was highest in the top soil and decreased with soil depth, indicating that C and N were consistently and positively correlated across the forest fragments.

Our study found that fine roots were distributed predominately in the top soil layer (0–20 cm) (Table 6). In addition, fine root distribution followed a pattern similar to that of soil C (Tables 5 and 6). These results are consistent with those of previous studies conducted in other forest ecosystems^40^. Our results showed that soil C concentration was positively correlated with fine roots in all soil layers and across all forest fragments. In addition, the ratios of soil C:N and fine root C:N showed edge effects. This may be because the forest edges altered the forest habitat, further affecting biogeochemical cycles^38^. Microclimatic changes that have been reported after fragmentation, such as increases in temperature, soil drying and the incidence of wind, affect soil C stocks in different ways^41^. Our results differed from a recent study which found significant increases in soil C stocks at the edge of forest fragments. Their study found that forest fragmentation may result in increased mortality of adult trees after about 30 years of isolation^29^.

## Conclusions

In this study, we characterized the spatial variation in C density for a C-rich forest ecosystem and investigated how patch size drove this variation. We found that C density of aboveground biomass, soil, and fine roots increased with patch size. Both soil C and aboveground biomass C were important contributors to total ecosystem C. The density of soil C, soil N, fine root C, and fine root N were highly heterogeneous among different fragments and soil depths. The components of ecosystem C density were all influenced by edge effects. Our results suggest that habitat fragmentation, and the resultant edge-affected habitats, drastically limit the capacity of forests to store C across human-modified landscapes. Results of this study are likely to be integrated into forest conservation strategies and used to evaluate C stocks at a landscape scale. This study also offers important data for developing and validating C density models in fragmented forests at a global scale.
